# Simultaneous determination of tylosin and josamycin residues in muscles, liver, eggs and milk by MLC with a monolithic column and time-programmed UV detection: application to baby food and formulae

**DOI:** 10.1186/1752-153X-8-37

**Published:** 2014-06-20

**Authors:** Jenny Jeehan Nasr, Shereen Shalan, Fathalla Belal

**Affiliations:** 1 Department of Pharmaceutical Analytical Chemistry, Faculty of Pharmacy, University of Mansoura, Mansoura 35516, Egypt

**Keywords:** Micellar liquid chromatography, Tylosin, Josamycin, Monolithic column, Chicken muscles, Eggs

## Abstract

**Background:**

Tylosin and Josamycin are macrolide antibiotics. They are used in the treatment of pneumonia, arthritis and mastitis in cattle, and mycoplasma infections in poultry. The incorrect use of antibiotics has lead to the presence of antibiotic residues in foods. The residues cause toxic effects on consumers.

**Results:**

A simple and sensitive method was optimized and validated for the analysis of tylosin and josamycin residues in food samples. Analytical separation was performed in less than 10 min using a RP C_18_ monolithic column with time-programmed UV detection at 287 nm and 232 nm and a micellar solution of 0.17 M sodium dodecyl sulphate, 14% methanol and 0.3% triethylamine in 0.02 M phosphoric acid buffered at pH 4 as the mobile phase. The method was fully validated in accordance with ICH guidelines. The micellar method was successfully applied to quantitatively determine tylosin and josamycin residues in spiked chicken muscles, chicken liver, bovine muscles, liver, milk and eggs. It was also extended to the determination of tylosin and josamycin residues in chicken-based baby food and baby formulae. The compounds were separated by a monolithic column which, on account of its particular structure, could bear higher flow rates than usually found for this kind of analysis. High extraction efficiency for tylosin and josamycin was obtained without matrix interference in the extraction process and in the subsequent chromatographic determination. No organic solvent was used during the pretreatment step. Hence, it is considered an interesting technique for “green” chemistry.

**Conclusion:**

The proposed method was validated and successfully applied for the determination of tylosin and josamycin residues in spiked chicken muscles, chicken liver, bovine muscles, liver, milk and eggs. It was also extended to the determination of tylosin and josamycin residues in chicken-based baby food and baby formulae.

## Background

Macrolide antibiotics are a very important class of antibacterial compounds widely used in human and veterinary practices for both therapeutic and prophylactic treatments [1]. They are active agents against Gram-positive and some Gram-negative bacteria [2]. Macrolides are also employed as growth promoters in stock farming in food producing animals [3].

**Figure 1:**
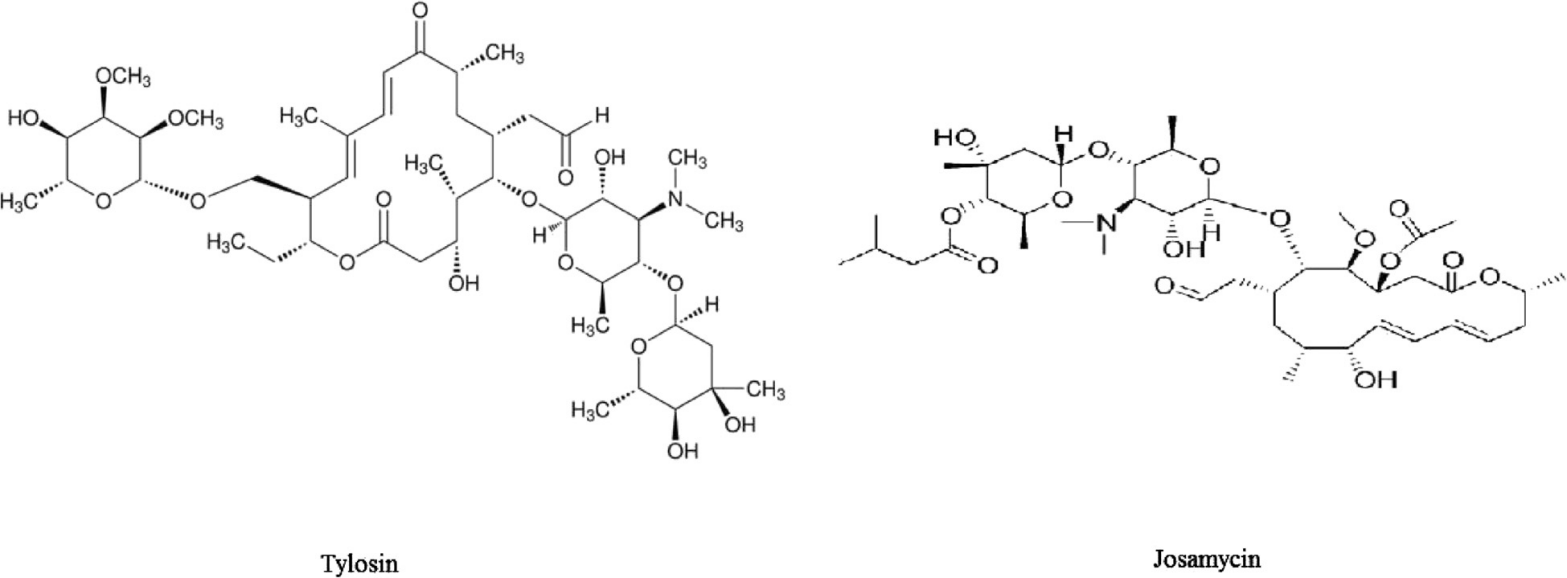
Chemical structures of the drugs investigated.

Tylosin (TS) and josamycin (JM) (Figure 1) belong to the class of 16-membered macrolide antibiotics [4]. They are used in the treatment of pneumonia, arthritis and mastitis in cattle, and mycoplasma infections in poultry [5,6]. The incorrect use of antibiotics and the disrespect for withdrawal time after treatment have lead to the presence of antibiotic residues in foods. The residues cause toxic effects on consumers, such as allergic reactions in sensitive or sensitized individuals, provoke the development of antibiotic-resistant bacteria and may induce cross-resistance against other antibiotics of similar structure or mechanism of action, making it more difficult to treat certain infections [7]. To ensure human food safety, the European Union (EU) has set maximal residue limits (MRLs) for some macrolides in tissues and eggs. EU has established a maximum residue limit of 100 μg/Kg of TS and 200 μg/Kg of JM in bovine and chicken tissues and 200 μg/Kg of each of TS and JM in eggs and 50 μg/Kg of TS in milk [8,9]. Therefore, simple and reliable analytical methods are required to monitor these drug residues in edible tissues of livestock animals.

There is no current legislation which establishes limits of veterinary drug residues in meat-based baby food and baby formulae. As a result of this lack of regulations for veterinary drugs, a zero-tolerance policy is applied for veterinary drug residues in baby food and formulae [10] which means that the presence of these compounds is illegal at any level. Therefore, the application of the “zero tolerance” concept requires the development of sensitive analytical methods to determine the presence of these compounds at very low concentration levels [11].

Literature revealed several HPLC methods for the simultaneous determination of TS and JM residues in muscle tissues. TS and JM residues were determined together with other macrolide antibiotics in meat [12], bovine, porcine and poultry muscles [13] using UV detection. They were also determined in sheep’s milk using UV-diode array detector [14]. Liquid chromatography coupled with electrospray ionization tandem mass spectrometry was used for the determination of TS and JM residues among other veterinary drugs in bovine, porcine and chicken muscles [15] and with other macrolide antibiotics in meat and fish [16]. HPLC [17,18] and UPLC [11] coupled with tandem mass spectrometry were also used.

Micellar liquid chromatography (MLC) allows complex matrices to be analyzed without the aid of extraction and with direct injection of samples [19]. Micelles tend to bind proteins competitively, thereby releasing protein-bound drugs and proteins, rather than precipitating into the column. Proteins are solubilized and washed harmlessly away, eluting with the solvent front. This means that costs and analysis times are cut considerably [20]. Micellar mobile phases usually need less quantity of organic modifier and generate less amount of toxic waste in comparison to aqueous-organic solvents, so that they are less toxic, non-flammable, biodegradable and relatively inexpensive [21]. Because of these advantages, MLC is considered an interesting technique for “green” chemistry that copes with current concern about the environment [22]. MLC has proved to be a useful technique in the determination of diverse groups of compounds in several matrices [23-25], including food samples [26,27].

Nowadays, the most challenging trend in liquid chromatography is the development of new sorbents, which are able to separate complicated substances efficiently. One of these novel types of sorbents is monolithic silica. They have a different structure compared to conventional silica [28]. Monolithic columns contain a special silica (or another material), which is not formed by particles. They are made by sol–gel technology, which enables formation of highly porous material, containing macropores and mesopores in its structure [28]. Due to these facts, higher flow rates can be used while the resolution of the silica rod column is much less affected in comparison to particulate materials after increasing the flow-rate and column back-pressure is still low. Another practical advantage is a short time needed for column equilibration when a mobile phase gradient is used [29]. There are a few works that deal with the practical application of monolithic columns in LC [30,31].

The present study describes, for the first time, a rapid, simple, sensitive and selective MLC–monolithic method with UV detection for the simultaneous determination of TS and JM residues in chicken muscles, chicken liver, bovine meat, liver, eggs and milk. The proposed procedure benefits from the two main advantages associated to the use of micellar mobile phases, namely, the use of environment-friendly eluents and fast and easy sample preparation. The procedure makes use also of the main advantage of monolithic columns which is the use of high flow rate without increasing the column back pressure. The procedure could also be extended to the analysis of these residues in chicken or meat-based baby food in addition to baby formulae.

### Experimental

#### Apparatus

Chromatographic analyses were carried out using a Shimadzu Prominence HPLC system (Shimadzu Corporation, Japan) with a LC-20 AD pump, DGU-20 A5 degasser, CBM-20A interface, and SPD-20A UV–VIS detector with 20 μL injection loop. The columns used were reversed-phase Chromolith® Performance (RP-18e, 100 mm × 4.6 mm i.d.) column obtained from Merck (Darmstadt, Germany) and Nucleodur MN-C18 column (150 mm × 4.6 mm i.d., 5 μm particle size), Macherey-Nagel, Düren, Germany. Centrifugation was carried out using TDL-60B Centrifuge (Anke, Taiwan). BHA-180 T Sonicator (Abbotta Corporation, USA) was used. Tissue homogenization was made using Tissue Master-125 Homogenizer (Omni International, Georgia, USA).

#### Reagents and materials

All reagents and solvents used were of HPLC grade. Tylosin tartrate and Josamycin analytical standard was of 100% purity from Sigma-Aldrich (Seelze, Germany). Methanol, *1*-propanol, acetonitrile and sodium dodecyl sulphate (SDS) were from Sigma-Aldrich (Seelze, Germany). Triethylamine and phosphoric acid were from Riedel-deHaën (Seelze, Germany). Regenerated cellulose membrane filters and syringe filters (Minisart RC25) with pore size 0.45 μm were from Sartorius-Stedim (Goettingen, Germany). Chicken muscles, chicken liver, bovine meat and liver samples and eggs were purchased from the local market. Milk was from Juhayna® (Cairo, Egypt) and Infant Formula Milk was from Hero® (Spain). Chicken noodles baby food, precooked, was from Gerber® (USA).

#### Preparation of solutions

Stock solutions of 0.2 mg mL^−1^ of each TS and JM were prepared by dissolving in methanol. Working solutions were prepared by diluting the stock solutions with the mobile phase. Stock solutions were found to be stable for one week if kept in the refrigerator protected from light.

#### Preparation of calibration curves

Working solutions containing 1 – 200 μg mL^−1^ of TS and 5 – 500 μg mL^−1^ of JM were prepared by serial dilutions of aliquots of the stock solutions. 20 μL aliquots were injected (triplicate) and eluted with the mobile phase under the reported chromatographic conditions. The average peak areas of each drug were plotted *versus* the concentrations of drug in μg mL^−1^. Alternatively, the corresponding regression equations were derived.

#### Analysis of bulk substance

The method mentioned under the previous section was applied to the determination of the purity of TS and JM raw materials. The percentage recoveries were calculated by referring to the calibration graphs previously prepared or by applying the regression equations.

#### Samples preparation

2.5 g of each of samples were accurately weighed and 5 mL of milk were measured and spiked with aliquots of TS and JM solutions. The spiked samples were mixed with 25 mL of 0.17 M SDS solution of pH 4. The solid samples were then homogenized at 5000 rpm for 5 min, then, the homogenate was sonicated for 15 min, and then centrifuged at 3000 rpm for 5 min. The egg and milk samples were not homogenized, but they were only sonicated for 2 min without centrifugation. The supernatant of all samples was filtered through 0.45 μm membrane filters using vacuum pump and diluted with the mobile phase. Aliquots of 20 μL were injected (triplicate) and eluted with the mobile phase under the reported chromatographic conditions.

## Results and discussion

**Figure 2:**
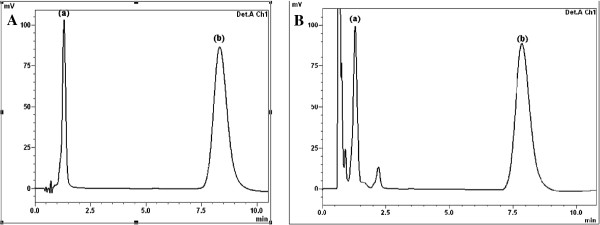
**Chromatogram showing: (a) 100 μg mL**^**−1 **^**TS, (b) 250 μg mL**^**−1 **^**JM in: (A) TS and JM standards. (B)** Baby Formula milk.

The proposed method permits the quantitation of TS and JM in raw materials, in chicken muscles, chicken liver, bovine meat, liver and eggs. Figure 2A shows a chromatogram indicating good resolution of TS (t_R_ = 1.4 min) and JM (t_R_ = 8.6 min). The proposed method offers high sensitivity: as low as 0.11 μg mL^−1^ of TS and 0.30 μg mL^−1^ of JM could be detected accurately.

### Selection and optimization of chromatographic conditions

To achieve the best chromatographic conditions, the mobile phase composition was optimized to provide sufficient selectivity and sensitivity in a short separation time. The different chromatographic conditions affecting the separation and resolution of TS and JM were carefully studied and optimized. The results of the optimization study are summarized in Table 1.

**Table 1:** Optimization of experimental factors affecting the chromatographic performance of the proposed method

**Parameter**	**Number of theoretical plates (N)**		**Tailing factor**		**Resolution (Rs)**
	**TS**	**JM**	**TS**	**JM**	**TS/JM**
Organic modifier nature					
Methanol	7566	12598	1.06	1.10	11.10
1-Propanol	5974	12154	1.16	0.20	16.40
Acetonitrile	4253	9098	1.10	0.10	14.10
Methanol concentration (%)					
8	3791	9496	1.05	1.09	9.97
10	5566	11598	1.06	1.10	11.10
12	4770	10204	1.17	1.24	11.42
14	7312	12723	1.09	1.10	11.16
16	4549	9675	1.21	1.31	11.80
SDS concentration (M)					
0.10	4317	8931	1.20	1.24	12.72
0.12	5800	9848	1.20	1.28	12.07
0.15	5312	10723	1.21	1.21	11.67
0.17	7516	12973	1.09	1.13	10.81
0.20	3429	10814	1.19	1.05	10.76
pH					
2.5	4247	9564	1.18	1.24	12.83
3.0	5985	10611	1.17	1.23	12.89
4.0	7516	12973	1.08	1.13	10.84
5.0	4830	11349	1.19	1.14	11.80

### Choice of column

Two different columns were tested for performance investigations, including: reversed-phase Chromolith® Performance C18 column, and Nucleodur MN-C18 column. The experimental studies revealed that the first column was more suitable, since it produced well-resolved peaks with a very high sensitivity within a reasonable analytical run time.

### Choice of appropriate detection wavelength and time program

The UV detector responses of TS and JM were carefully studied and the best wavelengths were found to be 287 nm and 232 nm for TS and JM, respectively showing the highest sensitivity. Programmable UV detection was employed to allow sensitive determination of both TS and JM simultaneously. TS was detected at 287 nm within 0–5 min, while, JM was detected at 232 nm after 5 min.

### Choice of a suitable flow rate

As the peak of JM was very late at flow rate 1 mL min^−1^, a flow rate of 2 mL min^−1^ was chosen in order to have a reasonable analytical run time (10 min) for the assay. The use of this considerably high flow rate was possible due to the use of a monolithic column which has the advantage of using high flow rates without affecting the column back pressure.

### Mobile phase composition

Several modifications in the micellar mobile phase composition were performed in order to study the possibilities of changing the selectivity of the chromatographic system. These modifications included the change of the concentration and type of organic modifier, the surfactant concentration, and the pH. The mobile phase was prepared using 0.3% triethylamine and 0.02 M phosphoric acid. The effect of changing the type of organic modifier on the selectivity and retention times of TS and JM was investigated using mobile phases containing 10% methanol, *1*-propanol or acetonitrile. Methanol was the best, giving well-resolved peaks and the highest number of theoretical plates. The effect of changing the concentration of organic modifier on the selectivity and retention times of TS and JM was investigated using mobile phases containing concentrations of 10–16% methanol and containing 0.15 M SDS and buffered at pH 4. 14% of methanol was the best, giving well-resolved peaks and the highest number of theoretical plates. Hence, a small amount of methanol is added to accelerate and control the elution of the drug. The effect of changing the concentration of surfactant on the selectivity and retention times of TS and JM was investigated using mobile phases containing SDS concentrations in the range 0.1–0.2 M and containing 14% methanol and buffered at pH 4. 0.17 M SDS was the best, giving well-resolved peaks and highest number of theoretical plates. The effect of changing the pH of the mobile phase on the selectivity and retention time of TS and JM was investigated using mobile phases of pH ranging from 2.5–5.0 with 0.17 M SDS concentration and 14% methanol. pH of 4.0 was most appropriate, giving well-resolved peaks and the highest number of theoretical plates. Values of pH higher than 5.0 resulted in very low number of theoretical plates.

After these experimental investigations, the assay was carried out using a Chromolith® Performance C18 column with mobile phase consisting of 0.17 M sodium dodecyl sulphate–14% methanol–0.3% triethylamine–0.02 M phosphoric acid at pH 4.0 and programmable UV detection with a flow rate of 2.0 mL min^−1^.

### System suitability test parameters

To ascertain the reproducibility of the MLC method, system suitability tests were performed using the working standard solutions of TS and JM. Resolution (Rs), theoretical plates number (N) and tailing factor (T) were measured as the criteria for system suitability testing. These results are satisfactory compared to the minimum values necessary for an acceptable method.

### Method validation

The validity of the proposed MLC method was tested in terms of linearity, ranges, limits of detection, limits of quantification, accuracy and precision.

#### Linearity and range

Under the above-described experimental conditions, linear relationships were established by plotting peak areas against TS and JM concentrations. The concentration ranges were found to be 1–200 μg mL^−1^ and 5–500 μg mL^−1^ for TS and JM, respectively. Linear regression analysis of the data gave the following equations:

$$\mathrm{TS}:\;P\;=\;-3.4\times 10^{3}+\;9.9\times 10^{3}C\;\left(r\;=\;0.9999\right)$$

$$\mathrm{JM}:\;P\;=\;1.2\times 10^{5}+\;1.3\times 10^{4}C\;\left(r\;=\;0.9999\right)$$

Where P is the peak area and C is the concentration of drug in μg mL^−1^ and r is the regression coefficient.

The high values of the correlation coefficients (r-values >0.999) indicate good linearity of the calibration graphs. Statistical analysis of the data gave small values of the % relative error, (% Er) 1.21% and 0.56% for both TS and JM, respectively [29].

#### Limit of quantitation (LOQ) and limit of detection (LOD)

The limit of quantitation (LOQ) was determined by establishing the lowest concentration that can be measured according to ICH Q2B recommendations [32] below which the calibration graph is non linear and was found to be 3.6 μg g^−1^ and 9.9 μg g^−1^ for TS and JM, respectively. The limit of detection (LOD) was determined by establishing the minimum level at which the analyte can be reliably detected (S/N = 3); it was found to be 1.1 μg g^−1^ (1.0 × 10^−7^ M) and 3.0 μg g^−1^ (3.6 × 10^−7^ M) for TS and JM, respectively.

#### Accuracy

The accuracy of analytical method is defined as the agreement of the results obtained by this method with the true values. To test the validity of the proposed method, it was applied to the determination of pure samples of TS and JM over the range of 1.0–200.0 μg mL^−1^ and 5.0–500.0 μg mL^−1^, respectively. The results obtained were in good agreement with those obtained using the comparison HPLC method [13]. Using Student’s t-test and the variance ratio F-test revealed no significant difference between the performance of the two methods regarding the accuracy and precision, respectively (Table 2) [33].

**Table 2:** Statistical analysis of the results obtained by the proposed and reference methods for pure samples of tylosin and josamycin

**Parameter**	**TS**		**JM**	
	**Proposed**	**Reference**	**Proposed**	**Reference**
% Recovery	90.4	97.5	104.3	98.7
	100.5	103.2	99.7	101.7
	99.0	98.8	100.8	99.3
	99.5		100.1	
	100.6		99.8	
	99.8		100.2	
	99.9		99.2	
	99.9		99.9	
Mean $\left(\bar{X}\right)$	98. 7	99.8	100.5	99.9
± S.D.	3.4	2.9	1.6	1.6
Variance	11.5	8.9	2.6	2.5
Students *t-*value^a^	0.62		0.61	
Variance ratio *F-*value^a^	1.37		1.04	

^a^Tabulated t- and F-values at p = 0.05 are: 2.26 and 4.74, respectively.

#### Precision

The intra-day precision was evaluated through replicate analysis of different concentrations of the two drugs pure forms within the specific working concentration ranges. Each sample was analyzed three successive times. Similarly, the inter-day precision was evaluated through replicate analysis of the three different concentrations on three successive days. The results obtained are summarized in Table 3. The data presented in Table 3 indicate high precision of the developed method. Good values of the average percentage recoveries and the small values of standard deviations indicate the high accuracy and precision, respectively.

**Table 3:** Accuracy and precision data for tylosin and josamycin using the proposed method

**Analyte**	**Concentration (μg mL**^**−1**^**)**	**Intra-day**^**a**^		**Inter-day**^**b**^	
		**Recovery (mean ± S.D.)**	**Er (%)**	**Recovery (mean ± S.D.)**	**Er (%)**
Tylosin	50.0	99.8 ± 0.2	0.1	99.1 ± 0.6	0.4
	100.0	99.5 ± 0.6	0.4	100.2 ± 0.4	0.2
	150.0	99.6 ± 0.6	0.3	99.7 ± 0.5	0.3
Josamycin	50.0	100.8 ± 0.3	0.2	100.3 ± 0.3	0.2
	125.0	99.6 ± 0.5	0.3	100.9 ± 0.4	0.2
	250.0	99.7 ± 0.5	0.3	99.6 ± 0.6	0.4

N.B. Each result is the average of three separate determinations.

^a^Intra-day: within the day.

^b^Inter-day: three consecutive days.

### Applications

**Figure 3:**
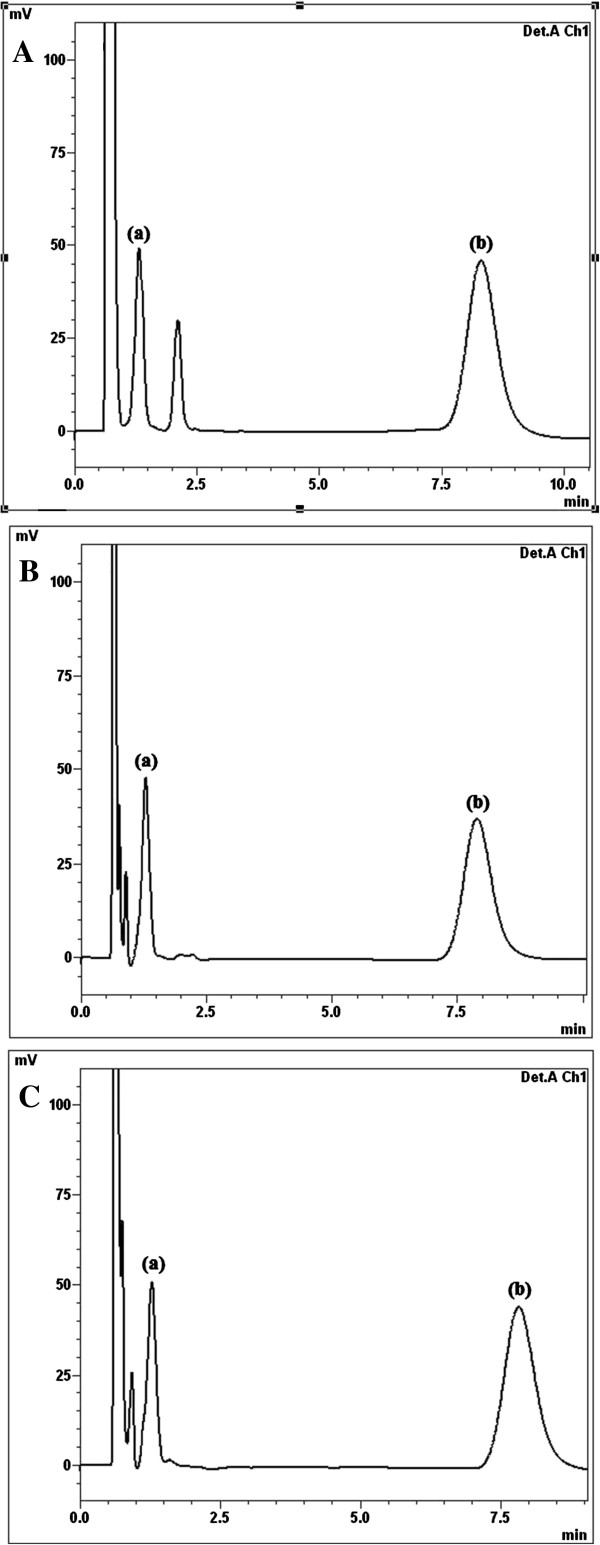
**Chromatograms showing: (a) 50 μg mL**^**−1**^**TS, (b) 125 μg mL**^**−1 **^**JM in: (A) Chicken liver. (B)** Eggs. **(C)** Milk.

The applicability of the procedure developed here to determine TS and JM was tested by analyzing it in spiked chicken muscles, liver, bovine meat, liver, eggs and milk in addition to spiked baby formula milk and chicken-based baby food. All samples were bought at a local supermarket. Table 4 shows the results of the analysis of TS and JM determined in all samples after homogenization with micellar solution, sonication, centrifugation and filtration. Samples were spiked at the following concentration levels: 50, 100 and 150 ppm for TS and 50, 125 and 250 ppm for JM. Three replicates of each concentration were injected into the chromatograph. The data obtained (Table 4) show satisfactory recoveries for TS and JM in all samples, and the results fall in the range of 98.00 – 102.60% and 97.80 – 101.13% for TS and JM, respectively.Figures 2B and 3 depict the chromatograms obtained from different spiked samples of TS and JM analyzed with the optimum mobile phase. These chromatograms reveal how a surveillance programme for TS and JM residues can be performed under the proposed chromatographic conditions. The low detection limits of the proposed method are useful for the determination of any traces of TS and JM residues that are prohibited in meat or chicken-based baby food and baby formula milk.

**Table 4:** Assay of tylosin and josamycin in food samples using the proposed and reference methods

**Method**	**TS**		**JM**		**TS**		**JM**	
	**Prop.**	**Ref.**	**Prop.**	**Ref.**	**Prop.**	**Ref.**	**Prop.**	**Ref.**
Sample type	Chicken muscle				Chicken liver			
Mean recovery $\left(\bar{X}\right)$^a^	101.10	100.62	101.13	100.86	101.93	99.13	99.97	98.47
± S.D.	0.72	0.82	2.71	2.17	2.01	2.29	3.16	2.84
Variance	0.52	0.67	7.34	4.7	4.04	5.24	9.96	8.04
Students *t-*value^b^	0.79		0.13		1.59		0.61	
Variance ratio *F-*value^b^	1.29		1.55		1.30		1.24	
Sample type	Bovine muscle				Bovine liver			
Mean recovery $\left(\bar{X}\right)$^a^	102.60	100.53	97.80	96.87	99.43	97.53	98.40	98.20
± S.D.	1.05	2.63	1.57	0.70	2.49	1.30	1.65	2.36
Variance	1.11	6.94	2.47	0.49	6.20	1.69	2.71	5.56
Students *t-*value^b^	1.26		0.94		1.17		0.12	
Variance ratio *F-*value^b^	6.25		5.01		3.66		2.05	
Sample type	Milk				Eggs			
Mean recovery $\left(\bar{X}\right)$^a^	99.53	98.87	99.40	98.93	100.93	100.60	100.80	100.70
± S.D.	2.35	2.81	2.61	2.58	3.02	2.43	2.72	1.87
Variance	5.52	7.89	6.84	6.65	9.14	5.92	7.41	3.51
Students *t-*value^b^	0.31		0.22		0.15		0.05	
Variance ratio *F-*value^b^	1.43		1.03		1.54		2.11	
Sample type	Baby food				Baby formulae			
Mean recovery $\left(\bar{X}\right)$^a^	100.03	99.70	99.67	98.03	98.00	97.03	98.80	97.37
± S.D.	2.51	1.90	3.07	1.98	2.85	1.91	1.35	1.80
Variance	6.30	3.61	9.40	3.94	8.13	3.64	1.83	3.24
Students *t-*value^b^	0.18		0.77		0.49		1.10	
Variance ratio *F-*value^b^	1.75		2.38		2.23		1.77	

^a^Number of experiments = 3.

^b^Tabulated *t*- and *F*-values at p = 0.05 are: 2.78 and 19.00, respectively.

## Conclusion

The proposed procedure is useful for food quality testing and control areas to determine the content of TS and JM in chicken muscles, liver, bovine muscles, liver, milk and egg samples. Moreover, it allows the detection of traces of TS and JM residues in meat or chicken-based baby food and baby formula milk with high sensitivity. One advantage of this procedure is the possibility of injecting the samples directly into the chromatographic system with no previous treatment other than homogenization, dilution and filtration, thus avoiding tedious extractions from matrices. Validation according to ICH regulation provides satisfactory results in terms of sensitivity, linearity, accuracy and recoveries and at the ppm level. It is noteworthy that the use of micellar mobile phases endows the procedure advantages such as non flammability, biodegradability and low cost. Current concern about the environment also reveals MLC as an interesting technique for “green” chemistry because it uses mobile phases containing low amounts of organic solvents. These micellar mobile phases have a low toxicity and are not producing hazardous wastes. It can be also concluded that, under our conditions, the monolithic column could operate at a higher flow rate than a conventional RP column with a reduced pressure and shorter washing and re-equilibration times.

## Abbreviations

TS: Tylosin; JM: Josamycin; MLC: Micellar liquid chromatography; LOD: Limit of detection; LOQ: Limit of quantification.

## Competing interests

The authors declare that they have no competing interests.

## Authors’ contributions

JJN proposed the subject, participated in the study design, assay design, literature review, conducted the validation of the assay, analysis of the samples, participated in the results discussion and participated in preparing the manuscript. ShSh participated in the study design, assay design, conducted the validation of the assay, analysis of the samples and participated in the results discussion. FFB designed the study, participated in the results discussion and revised the manuscript. All authors read and approved the final manuscript.
